# Estimation of 1 km Dawn–Dusk All-Sky Land Surface Temperature Using a Random Forest-Based Reanalysis and Thermal Infrared Remote Sensing Data Merging (RFRTM) Method

**DOI:** 10.3390/s25020508

**Published:** 2025-01-16

**Authors:** Yaohai Dong, Xiaodong Zhang, Xiuqing Hu, Jian Shang, Feng Zhao

**Affiliations:** 1 Committee of Science and Technology, Shanghai Academy of Space Technology, Shanghai 201109, China; dongyaohai@hotmail.com; 2 Shanghai Spaceflight Institute of TT&C and Telecommunication, Shanghai 201109, China; zhao_xiang_feng@163.com; 3 Key Laboratory of Radiometric Calibration and Validation for Environmental Satellites (KLRCV), National Satellite Meteorological Center, China Meteorological Administration, Beijing 100081, China; huxq@cma.gov.cn; 4 FengYun Meteorological Satellite Innovation Center, National Satellite Meteorological Center, China Meteorological Administration, Beijing 100081, China; shangjian@cma.gov.cn

**Keywords:** land surface temperature, all-sky LST, dawn–dusk time, thermal infrared remote sensing, reanalysis data, merging

## Abstract

All-sky 1 km land surface temperature (LST) data are urgently needed. Two widely applied approaches to derive such LST data are merging thermal infrared remote sensing (TIR)–passive microwave remote sensing (PMW) observations and merging TIR reanalysis data. However, as only the Moderate Resolution Imaging Spectroradiometer (MODIS) is adopted as the TIR source for merging, current 1 km all-sky LST products are limited to the MODIS observation time. Therefore, a gap still remains in terms of all-sky LST data with a higher temporal resolution or at other times (e.g., dawn–dusk time). Under this background, this study merged the observations of the Medium Resolution Spectrum Imager (MERSI-LL) on board the dusk–dawn-orbit Fengyun (FY)-3E satellite and Global Land Data Assimilation System (GLDAS) data to estimate dawn–dusk 1 km all-sky LST using a random forest-based method (RFRTM). The results showed that the model had good robustness, with an STD of 0.62–0.86 K of the RFRTM LST, compared with the original MERSI-LL LST. Validation against in situ LST showed that the estimated LST had an accuracy of 1.34–3.71 K under all-sky conditions. In addition, compared with the dawn–dusk LST merged from MERSI-LL and the Special Sensor Microwave Imager/Sounder (SSMI/S), the RFRTM LST showed better performance in accuracy and image quality. This study’s findings are beneficial for filling the gap in all-sky LST at high spatiotemporal resolutions for associated applications.

## 1. Introduction

Land surface temperature (LST), which characterizes the thermodynamic state between the Earth’s surface and the atmosphere, is a crucial driver of energy and water interactions in the Earth–atmosphere system [1,2,3]. Accordingly, it has been listed as an essential climate variable by the Global Climate Observing System of the World Meteorological Organization (WMO). The accurate measurement of LST is vital not only to various applications (e.g., drought early warning in agriculture [4,5,6], urban head island monitoring and land use investigation in environmental sciences [7,8,9,10], numerical weather prediction in meteorology [11,12,13,14], and so on) but also to studies associated with basic land surface processes such as climate change [15,16,17], energy balance and radiation budget [18,19,20], and the water and carbon cycles [21,22,23]. Satellite remote sensing is the only practical approach to rapidly map spatiotemporal continuous LST on regional and global scales, which is impossible when using ground-based measurements.

Satellite thermal infrared remote sensing (TIR) has been identified as a key method for acquiring LST. After nearly 50 years of development and improvements in the algorithms, TIR LST products—for example, the Terra/Aqua Moderate Resolution Imaging Spectroradiometer (MODIS) LST, the Meteosat second-generation Spinning Enhanced Visible and InfraRed Imager (MSG-SEVIRI) LST, and the Fengyun (FY)-3 Medium Resolution Spectrum Imager (MERSI) LST—now offer satisfactory accuracy and meet the requirements of public applications [24,25,26,27,28,29]. However, due to cloud effects, TIR is incapable of estimating LST under non-clear-sky conditions. As a consequence, the lack of cloudy-sky TIR LST obstructs the development of studies and applications in which all-sky LST is an indispensable input, making it an urgently needed resource [30,31].

Due to the penetrability of passive microwave remote sensing (PMW) in cloud conditions, it is considered practical to merge TIR and LST to derive an all-sky LST with moderate/high spatial resolutions (e.g., 1 km). Over the last decade, numerous methods of all-sky LST merged from TIR–PMW have been developed through (semi-)empirical [32,33,34,35], physical [36], and machine learning algorithms [37,38,39,40,41], and corresponding all-sky LST products have been released for various applications [42]. Unfortunately, gaps between the adjacent scanning swaths of current polar-orbit PMW sensors make for a considerable lack of brightness temperature (BT) at mid- to low-latitude regions [36,39]. As a consequence, either the TIR–PMW merged LST is spatially incomplete (i.e., not really all-sky available), or there remains potentially high uncertainty over low-latitude areas from gap-filling algorithms of PMW BT. Compared with PMW, the reanalysis data, such as the Global Land Data Assimilation System (GLDAS), the Modern-Era Retrospective Analysis for Research and Applications (MERRA), the National Centers for Environmental Prediction (NCEP), the China Land Data Assimilation System (CLDAS), and the 5th ECMWF Atmospheric Reanalysis (ERA-5), have the advantage of providing completely spatially seamless LST-related data. Therefore, with the various methods proposed, studies on estimating all-sky LST from merging TIR–reanalysis data have made rapid progress in recent years [43,44,45,46].

Existing all-sky LST products, whether merged from TIR–PMW or TIR–reanalysis data, can provide a spatial resolution of up to 1 km. Nevertheless, to the best of our knowledge, as the Terra/Aqua MODIS LST is the only applied TIR source utilized in these products, the times of these TIR–PMW merged data (i.e., MODIS Advanced Microwave Scanning Radiometer Earth Observing System (AMSR-E) and MODIS Advanced Microwave Scanning Radiometer 2 (AMSR2)) and the TIR–reanalysis (i.e., MODIS-GLDAS and MODIS-CLDAS) merged 1 km all-sky LST products are confined to the observation time of MODIS (i.e., local solar time around 10:30 or 1:30 at daytime/nighttime) [42]. Therefore, on one hand, current 1 km all-sky LST has not yet met the considerable demand of fields requiring high-temporal-resolution LST. On the other hand, a gap still remains in all-sky LST at other times, such as dawn–dusk-time LST, which has been reported as significant in applications such as real-time urban heat island dynamic monitoring [47,48], forest fire warning [49], daily soil freeze–thaw discrimination [50], and weather prediction [51], as well as models such as diurnal temperature cycle (DTC) reconstruction [52,53], land surface evaporation estimation [24,54,55], and soil moisture/temperature profile simulation [56,57].

In this context, the objective of this study was to fill the gap in the 1 km all-sky remotely sensed LST at dawn–dusk time using a practical method. Based on previous studies [58,59,60], this study proposed a random forest-based method—hereafter called the random forest-based reanalysis and TIR data merging (RFRTM) method—to estimate a dawn–dusk 1 km all-sky LST through merging FY-3E MERSI-LL and GLDAS data using a random forest-based model. Selecting the Tibetan Plateau as the study area, the model was trained using 18-month data, and the robustness of the model was validated by intercomparing the RFRTM LST with the original MERSI-LL LST in the subsequent year-long period. Then, the dawn–dusk RFRTM LST was validated against the in situ LST from ground sites over the area to assess its performance under all-sky conditions. Coupled with previous studies on all-sky LST estimation, this study would be valuable for providing 6 h daily all-sky LST datasets, which will be of great benefit to associated studies and applications.

## 2. Materials

### 2.1. Study Area

**Figure 1:**
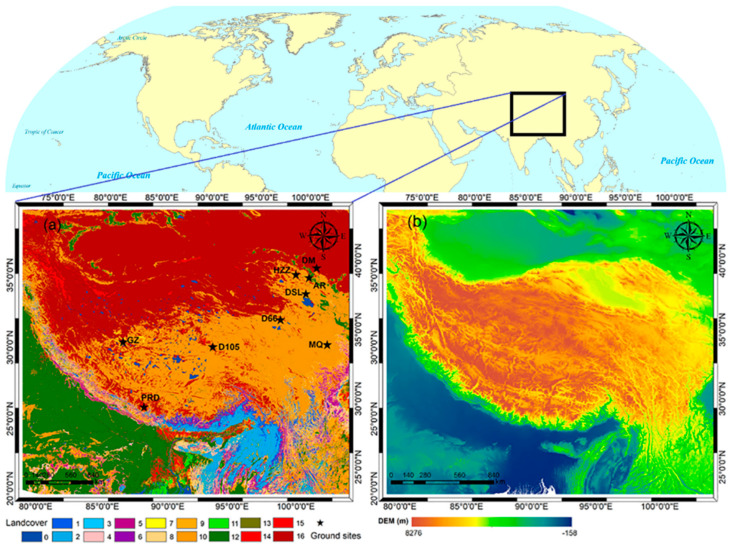
Land cover (**a**) and digital elevation model (DEM) (**b**) of the study area. The land cover is from MODIS MCD12Q1 products (https://earthdata.nasa.gov/, accessed on 1 November 2024)). The DEM is from the Shuttle Radar Topography Mission of Consortium for Spatial Information (SRTM-CSI) Web (http://srtm.csi.cgiar.org/, accessed on 1 November 2024)). The land cover codes are based on the International Geosphere–Biosphere Programme (IGBP) global classification scheme: 0, water; 1, evergreen needleleaf forest; 2, evergreen broadleaf forest; 3, deciduous needleleaf forest; 4, deciduous broadleaf forest; 5, mixed forest; 6, closed shrubland; 7, open shrubland; 8, woody savanna; 9, savanna; 10, grassland; 11, permanent wetland; 12, cropland; 13, urban and built-up; 14, cropland–nature vegetation; 15, snow and ice; and 16, barren or sparsely vegetated. Ground sites where in situ LST is measured are also shown (black stars).

The study area covered the Tibetan Plateau in southwestern China and its surrounding area. As shown in Figure 1, the Tibetan Plateau, with an average elevation of over 4000 m, is regarded as “the roof of the world” and “the water tower of Asia.” The unique location and complex topography of the area determine not only its diverse land covers and various climate zones but also its pivotal role in climate change in Asia and even the Northern Hemisphere [61,62]. As reported in previous studies, the rapid increase in LST over this area during the past two decades is highly related to, or can partly explain, the exacerbation of global warming, which has aroused wide concern within scientific communities [62,63,64,65]. Therefore, it is currently imperative to acquire all-sky LST data with a moderate/high spatial resolution over this area.

### 2.2. Data

#### 2.2.1. Remote Sensing Data

The remote sensing data included the FY-3E MERSI-LL L1 radiance product, the Advanced Spaceborne Thermal Emission and Reflection Radiometer (ASTER) global surface emissivity (GED) product (version 3), the MODIS Normalized Different Vegetation Index (NDVI) product, the MODIS albedo product (MCD43A3), and digital elevation model (DEM) data.

The FY-3E MERSI-LL L1 aggregated radiance product, which was from the Fengyun Satellite Data Center (http://satellite.nsmc.org.cn/, accessed on 1 November 2024) of the China Meteorological Administration (CMA), contained the radiance and surface emissivity from two TIR split-window bands (band 6 and band 7) with a 1000 m spatial resolution aggregated from the original 250 m resolution. The temporal resolution of these data was 12 h, which was consistent with the observation time of MERSI-LL (i.e., ~5:40 and ~17:40 local solar time).

ASTER GED (v3) was from the Earthdata website (https://earthdata.nasa.gov/, accessed on 1 November 2024) and contained a global surface emissivity of ASTER TIR bands from 2000 to 2008. The data had a spatial resolution of 100 m.

The MODIS NDVI and albedo product were also from the Earthdata website (https://earthdata.nasa.gov/, accessed on 1 November 2024). The selected NDVI product (MOD13A2) had a 16-day 1 km spatiotemporal resolution, while the albedo product (MCD43A3) was composed of daily 1 km data.

#### 2.2.2. Reanalysis Data

The reanalysis data used in this study were Global Land Data Assimilation System (GLDAS) data obtained from the Earthdata website (https://earthdata.nasa.gov/, accessed on 1 November 2024) GLDAS is a land surface process model that assimilates satellite observation and ground measurements and can provide the optimal estimation of global land surface state (e.g., wind speed profile, soil temperature and moisture profiles, snow depth, and water equivalent) and flux (e.g., sensible heat flux) variables [66,67]. In this study, nine geophysical variables including net longwave flux, downwelling longwave radiation, soil moisture (0–10 cm), canopy surface water, snow depth, water equivalent, soil temperature (skin surface, 0–10 cm), wind speed, and air temperature were used for implementing the proposed merging method. Section 3.2.2 gives the principle of selection of these variables. All the variables were derived from the GLDAS Noah products V2.1 (GLDAS_NOAH025_3H) with 3-hourly 0.25° (~25 km) spatiotemporal resolution.

#### 2.2.3. Atmospheric Profile Data

Thermodynamic Initial Guess Retrieval (TIGR) was used in this study as the atmospheric profile dataset. TIGR (Version 2002) was constructed by the Laboratoire de Meteorologie Dynamique (https://www.aeris-data.fr/en/projects/thermodynamical-initial-guess-retrieval-tigr/, accessed on 15 November 2024) and represented a worldwide set of atmospheric situations (2311 radiosonde reports) from polar to tropical atmospheres. Each profile is described from the surface to the top of the atmosphere by the values of temperature, water vapor, and ozone concentrations on a given pressure grid.

#### 2.2.4. Ground Measurements

In this study, surface longwave radiation measured at nine ground sites shown in Figure 1 was selected to measure the in situ LST for the validation of the estimated all-sky LST. In the Tibetan Plateau, there were five sites including D66, D105, Gaize (GZ), Maqu (MQ), and Pyramid (PRD); the corresponding data were from the Enhanced Observing Period (CEOP) of the Asia–Australia Monsoon Project (CAMP) on the Tibetan Plateau (CAMP/Tibet); the data on the other four sites in the Heihe River Basin at the northeast of the plateau—namely, Arou (AR), Daman (DM), Dashalong (DSL), and Huazhaizi (HZZ)—were from the Heihe Integrated Observatory Network [68,69]. The detailed site information including the location, altitude, land cover type, instrument, field of view (FOV), and data sampling interval are listed in Table 1. Contaminated longwave radiation data due to short-time disturbances such as instantaneous cloud-induced shadows were removed when the data value deviated by more than three times that of the standard deviation from the one-hour average. Then, the longwave radiation was converted to LST according to the following equation:

(1) $$T_{s\text{-}\mathrm{in}\mathrm{situ}}={\left(\frac{F_{u}^{l}-(1-\varepsilon )F_{d}^{l}}{\varepsilon \sigma }\right)}^{1/4}$$

where $F_{u}^{l}$ is the longwave upward radiation; $F_{d}^{l}$ is the longwave downward radiation; *σ* is land surface broadband emissivity, which is provided in the in situ data of each station; and *ε* is the Stefan–Boltzmann constant. Finally, two converted in situ LSTs at the time closest to the observation time of the FY-3E MERSI-LL were selected and linearly interpolated to the observation time of the satellite to obtain a relatively true LST for validation [70].

## 3. Methods

### 3.1. Retrieval of Dawn–Dusk 1 km TIR LST

In this study, the method developed in [58] was utilized to retrieve the TIR LST from the FY-3E MERSI-LL L1 radiance product. The method was developed based on the generalized split-window (GSW) algorithm [71], which can be expressed as follows (hereafter, the method in [58] is called GSW-M):

(2) $$\left\{\begin{array}{l} T_{s}=a_{0}+(a_{1}+a_{2}\frac{1-\varepsilon }{\varepsilon }+a_{3}\frac{\Delta \varepsilon }{\varepsilon^{2}})\frac{T_{6}+T_{7}}{2}+(a_{4}+a_{5}\frac{1-\varepsilon }{\varepsilon }+a_{6}\frac{\Delta \varepsilon }{\varepsilon^{2}})\frac{T_{6}-T_{7}}{2}\\ \varepsilon =(\varepsilon_{6}+\varepsilon_{7})/2\\ \Delta \varepsilon =\varepsilon_{6}-\varepsilon_{7} \end{array}\right.$$

where *T*_s_ is LST; *T*_6_ and *T*_7_ are the bright temperatures (BTs) of MERSI-LL bands 6 and 7, respectively; *ε*_6_ and *ε*_7_ are the surface emissivity of bands 6 and 7, respectively; ε is the mean surface emissivity of the two bands; Δ*ε* is the surface emissivity difference; and *a*_0_–*a*_6_ are the algorithm coefficients.

The implementation of the method is briefly summarized in five sections as follows:

(1) Estimation of MERSI-LL BT from the original MERSI-LL L1 data

In this section, at first, the radiance of bands 6 and 7 of MERI-LL was converted to efficient blackbody temperature using the Planck function. Then, the efficient blackbody temperature was converted to the BTs of the bands using the linear equation given in the data user guide (https://img.nsmc.org.cn/PORTAL/NSMC/DATASERVICE/OperatingGuide/FY3E/FY-3E_L1_Data_Instruction_MERSI-LL.pdf, accessed on 15 November 2024).

(2) Estimation of the land surface emissivity (LSE) of MERSI-LL

In this section, at first, the soil emissivity of ASTER bands was estimated from the mean emissivity of the ASTER band in the ASTER GED product and vegetation emissivity, which can be calculated from the vegetation emissivity spectra in the ASTER spectral library and the channel response function. Then, the MERSI-LL channel LSE (i.e., *ε*_6_ and *ε*_7_) was estimated from the ASTER channel LSE using a linear regression function.

(3) Estimation of the water vapor content (WVC)

In this section, the WVC was estimated from the BTs of MERSI bands and view zenith angle (VZA) using a system of empirical nonlinear equations.

(4) Simulation of observed MERSI BT

In this section, at first, the LST of a valid range, the mean emissivity of the MERSI-LL bands of a valid range (i.e., *ε*), the emissivity difference in the MERSI-LL bands (i.e., Δ*ε*) of a valid range, the WVC of a valid range, and the VZA of a valid range were all divided into several sub-ranges. Each sub-range of the above variables was combined as a simulation case. For each case, with the variation in variables with certain steps, numerous simulated MERSI BTs were generated using MODTRAN, which is a widely adopted radiative transfer model used to accurately simulate the observed BT for TIR sensors.

(5) Estimation of MERSI-LL LST

In this section, with plenty of simulated BTs and the corresponding LST from (4), the GSW coefficients in Equation (2) (i.e., *a*_0_–*a*_6_) can be determined using a stepwise regression. It should be noted that there were plenty of GSW coefficients corresponding to each case generated in (4). Therefore, a look-up table of GSW coefficients was established. Finally, with the actual observed MERSI BT estimated from (1), the actual mean emissivity of the MERSI-LL bands from (2), the emissivity difference in the MERSI-LL bands from (2), the actual VZA from (3), and the GSW coefficients in the corresponding case, the MERSI-LL LST was estimated using a stepwise iteration. The detailed theory and implementation of the MERSI-LL LST retrieval method are provided in [58].

However, it should be noted that this study focused on the Tibetan Plateau, while the referenced study [58] was focused on global conditions. Therefore, the following details for implementing the method should be adjusted to determine the appropriate coefficients of the GSW algorithm and the subsequent valid and reliable MERSI-LL LST over this area:

(1) In [58], global atmospheric profile data were used, while in this study, only tropical and mid-latitude area profiles were used.
(2) In [58], simulated BT was used when the MERSI VZA exceeded 60°, while in this study, MERSI L1 data with a VZA over 60° were removed due to possible large retrieval errors under this condition [26].
(3) In [58], when simulating BT using MOTRAN, the LST was divided into five sub-ranges: *T*_s_ ≤ 280 K, [275 K, 295 K], [290 K, 310 K], [305 K, 325 K], and *T*_s_ ≥ 320 K. The range of mean LSE of the two MERSI TIR bands was divided into two sub-ranges: [0.90, 0.96] and [0.94, 1.0]. In this study, the LST range was determined from statistics of MERSI-LL 1 km LST in 2023 over the study area (the data were from Fengyun Satellite Data Center (http://satellite.nsmc.org.cn/, accessed on 1 November 2024). The range of mean LSE of the two MERSI TIR bands was determined from the statistics of 1 km surface emissivity in the FY-3E MERSI-LL L1 product in 2023 over the study area. As a consequence, the divided LST sub-ranges in this study were as follows: *T*_s_ ≤ 260 K, [260 K, 280 K], [275 K, 295 K], [290 K, 310 K], [305 K, 325 K], and *T*_s_ ≥ 325 K. The range of mean LSE of the two MERSI TIR bands was divided into the following sub-ranges: [0.72, 0.80], [0.78, 0.86], [0.84, 0.92], and [0.90, 1.0].

### 3.2. Estimation of 1 km All-Sky LST Merging TIR LST and Reanalysis Data

#### 3.2.1. Basic Theory

It has been demonstrated in previous studies that a fine-resolution LST time series can be accurately retrieved using machine learning regression by combining time series of multiple coarse-resolution LST descriptors (e.g., PMW BT from different channels), as long as the number of LST descriptors and the length of the time series are sufficient. The basic principle is described as follows.

**Figure 2:**
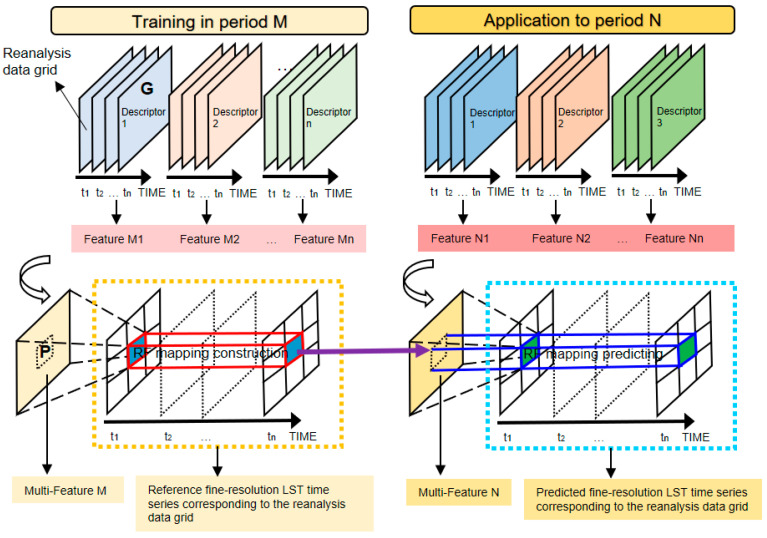
Illustration of (1) construction of a random forest-based mapping transform between time series of coarse-resolution LST descriptors in a reanalysis grid G and the referenced LST time series of a fine-resolution pixel P over a period M and (2) the application of the mapping transform to another period N. Note that not all fine-resolution subpixels in a coarse-resolution reanalysis grid are shown in the figure for the convenience of display.

As shown in Figure 2, suppose there is a coarse-resolution (e.g., 0.25°) reanalysis grid G and a fine-resolution (e.g., 1 km) pixel P located in the grid G. In the temporal dimension, within a given period M, the LST time series of P is capable of representing the surface thermodynamic temporal feature of the pixel-covered area. Meanwhile, despite the mismatch with respect to the spatial resolution between P and G, such a feature can be partly described by a time series of LST-related variables of G. Therefore, as long as sufficient LST descriptors from G are available, the temporal feature domain of LST, which consists of these descriptors, has the ability to fully explain the LST time series of P with a certain mapping transform. The mapping transform is expected to meet the following two criteria: (1) it is able to construct the exclusive link between the time series of multiple coarse-resolution LST descriptors and fine-resolution LST and (2) as long as the number of samples in M is sufficient, the mapping transform is expected to have a stable structure and be well generalized for predicting a new LST time series of P when applied to another period N (e.g., the mapping established under clear-sky LST of P can be generalized under cloudy-sky conditions of P with an acceptable accuracy). According to previous studies, machine leaning regression methods such as artificial neural networks (ANNs), support vector machines (SVMs), and random forests (RFs) can meet these requirements [37,39,72]. According to our preliminary study, among all current methods, the random forest regression has the best ability to construct the mapping transform in terms of generalized ability and resistance to overfitting [73]. Therefore, the clear-sky LST time series of P and time series of LST descriptors in G can be expressed as follows:

(3) $$\begin{array}{l} T_{s\text{-}\mathrm{TIR}\text{-}P}=RF_{T\text{-}P}(R_{\mathrm{clr}})\\ \mathrm{where}\\ T_{s\text{-}\mathrm{TIR}\text{-}P}=[T_{s\text{-}P}(t_{d\text{-}\mathrm{clr}\text{-}1}),T_{s\text{-}P}(t_{d\text{-}\mathrm{clr}\text{-}2}),\ldots ,T_{s\text{-}P}(t_{d\text{-}\mathrm{clr}\text{-}n})]\\ R_{\mathrm{clr}}=[D_{T\text{-}1\text{-}\mathrm{clr}}\;D_{T\text{-}2\text{-}\mathrm{clr}}\;\ldots \;D_{T\text{-}m\text{-}\mathrm{clr}}]T\\ \;\;\;=\left[\begin{array}{lll} d_{T\text{-}1}(t_{d\text{-}\mathrm{clr}\text{-}1}) & d_{T\text{-}1}(t_{d\text{-}\mathrm{clr}\text{-}2}) & \ldots \;\;d_{T\text{-}1}(t_{d\text{-}\mathrm{clr}\text{-}n})\\ d_{T\text{-}2}(t_{d\text{-}\mathrm{clr}\text{-}1}) & d_{T\text{-}2}(t_{d\text{-}\mathrm{clr}\text{-}2}) & \ldots \;\;d_{T\text{-}2}(t_{d\text{-}\mathrm{clr}\text{-}n})\\ \ldots & \;\;\ldots & \;\;\;\;\ldots \\ d_{T\text{-}m}(t_{d\text{-}\mathrm{clr}\text{-}1}) & d_{T\text{-}m}(t_{d\text{-}\mathrm{clr}\text{-}2}) & \ldots d_{T\text{-}m}(t_{d\text{-}\mathrm{clr}\text{-}n}) \end{array}\right] \end{array}$$

where **R**_clr_ denotes the matrix including vectors of LST descriptors’ time series of a reanalysis data grid containing the pixel P, **D**_T-*m*_ denotes the *m*th LST descriptors’ time series, and **RFT**_T-P_ denotes the RF-based mapping transform from the multiple LST descriptors’ time series of the G to the LST time series of P. Therefore, by applying this equation to clear-sky and cloudy-sky conditions, the initial 1 km all-sky LST can be expressed as follows:

(4) $$\begin{array}{l} {\hat{T}}_{s\text{-}\mathrm{RFT}\text{-}P}=\left\{\begin{array}{l} {\hat{T}}_{s\text{-}\mathrm{RFT}\text{-}\mathrm{clr}\text{-}P}=RF_{T\text{-}P}(R_{\mathrm{clr}})\;(a)\;\\ {\hat{T}}_{s\text{-}\mathrm{RFT}\text{-}\mathrm{cld}\text{-}P}=RF_{T\text{-}P}(R_{\mathrm{cld}})\;(b) \end{array}\right.\\ \mathrm{where}\;\\ R_{\mathrm{cld}}=[D_{T\text{-}1\text{-}\mathrm{cld}}\;D_{T\text{-}2\text{-}\mathrm{cld}}\;\ldots \;D_{T\text{-}m\text{-}\mathrm{cld}}]T\\ \;\;\;=\left[\begin{array}{lll} d_{T\text{-}1}(t_{d\text{-}\mathrm{cld}\text{-}1}) & d_{T\text{-}1}(t_{d\text{-}\mathrm{cld}\text{-}2}) & \ldots \;\;d_{T\text{-}1}(t_{d\text{-}\mathrm{cld}\text{-}n})\\ d_{T\text{-}2}(t_{d\text{-}\mathrm{cld}\text{-}1}) & d_{T\text{-}2}(t_{d\text{-}\mathrm{cld}\text{-}2}) & \ldots \;\;d_{T\text{-}2}(t_{d\text{-}\mathrm{cld}\text{-}n})\\ \ldots & \;\;\ldots & \;\;\;\;\ldots \\ d_{T\text{-}m}(t_{d\text{-}\mathrm{cld}\text{-}1}) & d_{T\text{-}m}(t_{d\text{-}\mathrm{cld}\text{-}2}) & \ldots d_{T\text{-}m}(t_{d\text{-}\mathrm{cld}\text{-}n}) \end{array}\right] \end{array}$$

where $\hat{T}$**_s_**_-RFT-clr-P_ and $\hat{T}$_s-RFT-cld-P_ denote the initial estimated 1 km LST of pixel P under clear-sky and cloudy-sky conditions, respectively, and *t*_d-cld-*n*_ denotes the *n*th cloudy-sky day pixel P.

The systematic error of the initial estimated LST time series from Equation (4) with respect to the original TIR LST time series of pixel P is as follows:

(5) $$\Delta e_{\mathrm{sT}\text{-}P}={\bar{\hat{T}}}_{s\text{-}\mathrm{RFT}\text{-}\mathrm{clr}\text{-}P}-{\bar{T}}_{s\text{-}\mathrm{TIR}\text{-}P}$$

Thus, the final estimated all-sky LST of pixel P can be obtained by optimizing the initial clear-sky and cloudy-sky LST, respectively, as follows:

(6) $$T_{s\text{-}\mathrm{RFT}\text{-}P}=\left\{\begin{array}{l} T_{s\text{-}\mathrm{RFT}\text{-}\mathrm{clr}\text{-}P}={\hat{T}}_{s\text{-}\mathrm{RFT}\text{-}\mathrm{clr}\text{-}P}-\Delta e_{\mathrm{sT}\text{-}P}\;(a)\\ T_{s\text{-}\mathrm{RFT}\text{-}\mathrm{cld}\text{-}P}={\hat{T}}_{s\text{-}\mathrm{RFT}\text{-}\mathrm{cld}\text{-}P}-\Delta e_{\mathrm{sT}\text{-}P}\;(b) \end{array}\right.$$

#### 3.2.2. Selection of LST Descriptors

In this study, LST descriptors were selected based on the theory of the land surface radiation budget, in which the longwave net radiation is expressed by the following equation [74]:

(7) $$R_{n}^{l}=\varepsilon F_{d}^{l}-\sigma \varepsilon T_{s}^{4}$$

where $R_{n}^{l}$ is net longwave radiation. Hereby, the LST can be derived as follows:

(8) $$T_{s}={\left(\frac{R_{n}^{l}-\varepsilon F_{d}^{l}}{\sigma \varepsilon }\right)}^{\frac{1}{4}}$$

The equation indicates that the LST can be functionally expressed by the net longwave radiation, the longwave downward radiation, and the land surface broadband emissivity. Among these descriptors, the former two are obtainable from the GLDAS data, while the all-sky land surface emissivity is unavailable. Therefore, four types of variables from GLDAS and MODIS data (i.e., the soil moisture at 0–10 cm depth, canopy surface water, and snow depth water equivalent from GLDAS and MODIS NDVI) were selected to describe the land surface emissivity [75,76,77]. In addition, four types of additional LST-related variables (i.e., wind speed, soil temperature profile at the surface and 0–10 cm depth, and air temperature from GLDAS data and MODIS albedo) were selected as ancillary descriptors to enhance the generalized ability of the RF-based mapping transform in this algorithm. Based on the above, the selected descriptors of LST in Equations (3) and (4) are listed in Table 2.

### 3.3. Implementation of the Method

**Figure 3:**
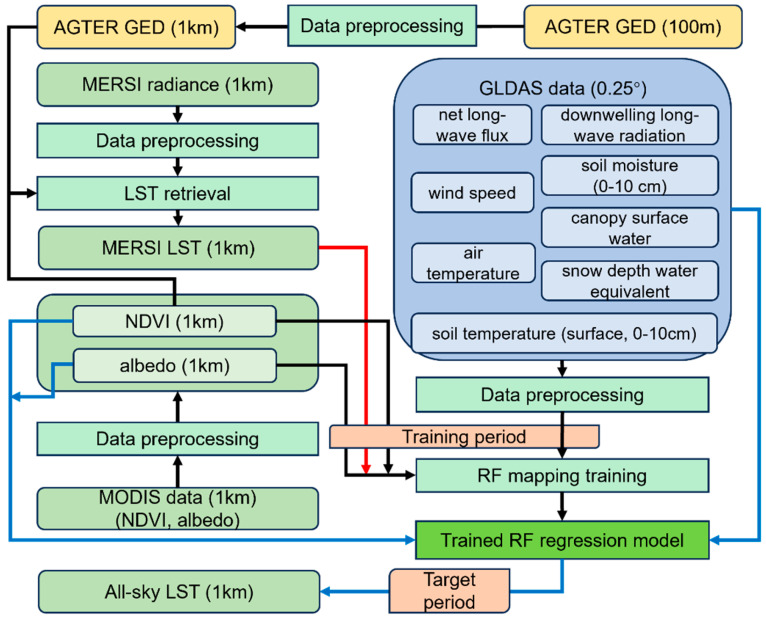
Flowchart of implementation of the method.

As shown in Figure 3, the method was implemented with the following stages: (1) TIR LST retrieval, (2) data preprocessing, (3) RFRTM model training, and (4) estimation of the 1 km all-sky RFRTM LST. In this study, the RFRTM model was trained using data from 1 April 2022 to 30 September 2023 (hereafter called the training period) to guarantee a sufficient sample size, and the model was applied over a period spanning from 1 October 2023 to 30 September 2024 (hereafter called the target period) to estimate the dawn–dusk 1 km all-sky LST merging the FY-3E MERSI-LL LST and GLDAS data.

Stage I: TIR LST retrieval

First, good-quality MERSI-LL TIR radiance was selected according to the quality assurance flag in the data fields and then converted to TIR band BT based on the conversion formula given in the product user guide (https://img.nsmc.org.cn/PORTAL/NSMC/DATASERVICE/OperatingGuide/FY3E/FY-3E_L1_Data_Instruction_MERSI-LL.pdf, accessed on 1 November 2024). Then, the 1 km daily MERSI-LL LST was retrieved from the BT, ASTER GED, and MODIS NDVI based on the GSW algorithm, as described in Section 3.2.1.

Stage II: Data preprocessing

This stage included the following steps:

(1) Temporally interpolate 16-day 1 km MODIS NDVI to daily resolution as follows:
  (9) $$NDVI_{i}(t)=a_{i}+b_{i}(t-t_{i})+c_{i}{\left(t-t_{i}\right)}^{2}+d_{i}{\left(t-t_{i}\right)}^{3}$$
  where *NDVI_i_*(*t*) is the resultant NDVI value at time t in the *i*th period, *t* is the interpolating point (i.e., the day of year (DOY) when NDVI is missing) between *t_i_* and *t_i_*_+1_, and *a*–*d* are cubic spline function parameters decided by the DOY and 16-day NDVI matrix in the *i*th period and the (*i* + 1)th period. The spline function is used here as it reduces both computational requirements and numerical instabilities arising with higher degree curves [78]. Then, considering the difference in observation time and spectral response function between MODIS and MERSI-LL, the MERSI-LL NDVI is converted from MODIS NDVI with an empirical method as follows [79]:
  (10) $$NDVI_{\mathrm{MERSI}\text{-}\mathrm{LL}}=\frac{1.98NDVI_{\mathrm{MODIS}}-0.07}{0.96NDVI_{\mathrm{MODIS}}+1.04}$$
(2) For a single 1 km MERSI-LL pixel M, select the spatially nearest GLDAS grid as its spatially matched GLDAS grid. Then, temporally interpolate the GLDAS data to the observation time of MERSI-LL using the cubic spline function as follows [43,44]:
  (11) $$D_{{G\text{-}M\text{-}t}_{M}}=SP(D_{G\text{-}M\text{-}t_{1}},\;D_{G\text{-}M\text{-}t_{2}},\;D_{G\text{-}M\text{-}t_{3}},\;D_{G\text{-}M\text{-}t_{4}})$$
  where **D**_G-M-tM_ is data of the GLDAS grid spatially matched with M and temporally interpolated for the observation of M; **D**_G-M-t1_, **D**_G-M-t2_, **D**_G-M-t3_, and **D**_G-M-t4_ are data of the GLDAS grid at four time points closest to the observation of M (*t*_1_ < *t*_2_ ≤ *t*_M_ < *t*_3_ < *t*_4_); and **SP** is the spline function. Then, the GLDAS data are spatiotemporally matched with the MERSI-LL data. Given that a GLDAS grid spatially contains a number of MERSI-LL pixels with different observation times, for different MERSI-LL pixels spatially matched with the same GLDAS grid, the corresponding interpolated GLDAS data can be different.
(3) Fill the missing 1 km albedo caused by cloud coverage with the statistics-based temporal filter [80].

Stage III: RFRTM model training

This stage included the following steps:

(1) For a single 1 km MERSI-LL pixel, train the RF regression mapping between the MERSI-LL LST and the LST descriptors from GLDAS data over the training period using Equation (3).
(2) Estimate the systematic error between the initial all-sky LST and the MERSI-LL LST via Equation (5).

Stage IV: all-sky LST estimation

This stage included the following steps:

(1) Estimate the initial all-sky 1 km LST (i.e., $\hat{T}$_s-RFT-P_) using Equation (4) over the target period.
(2) Estimate the final all-sky 1 km LST using Equation (6) over the target period.
(3) Repeat steps (1)–(4) pixel by pixel until all 1 km pixels are processed.

### 3.4. Evaluation of the RFRTM LST

The estimated 1 km RFRTM LST data were first intercompared with the 1 km original MERSI-LL LST data over the target period to examine the robustness of the model; then, the LST was validated against in situ LSTs under both clear-sky and cloudy-sky cases to evaluate the generalized ability of the model under all-sky conditions.

For the purpose of comparing the difference between the dawn–dusk 1 km all-sky LST from merging TIR–reanalysis data and that from merging TIR–PMW data, the 1 km all-sky LST merging FY-3E MERSI-LL and DMSP-F18 Special Sensor Microwave Imager/Sounder (SSMI/S) data, which were also based on a random forest-based model proposed in [39], were also estimated (hereafter termed the Zhang LST) and validated against the ground measurements. The coefficients of determination (*R*^2^), standard deviation (STD), mean bias error (MBE), and root mean square error (RMSE) were selected as the evaluation metrics.

In addition, the image quality of the RFRTM LST and the Zhang LST, i.e., the visual consistency of the RFRTM/Zhang LST and the original MERSI-LL LST, were evaluated according to the simple yet flexible index (SIFI) [81]. The SIFI was determined as follows:

(12) $$SIFI=\frac{\sqrt{E{\left(T_{E}-T_{T}\right)}^{2}}}{\sqrt{E{\left(T_{T}-T_{N}\right)}^{2}}}$$

where *E*(·) denotes the expectation function, *T*_E_ is the temperature image under evaluation, *T*_N_ is the temperature image at the native spatial resolution, and *T*_T_ is the temperature image at the target resolution. Lower SIFI meant the image under evaluation had more pixels with closer values and was visually consistent with the reference image. Therefore, a lower SIFI indicated a better image quality of the evaluated image, and vice versa.

Equation (12) was first applied to the daily RFRTM LST image using a daily MERSI-LL LST image as *T*_T_, while the MERSI-LL LST up-scaled to the native resolution of GLDAS (i.e., 0.25°) was used as *T*_N_. The SIFI of the Zhang LST was also calculated to compare with the SIFI of the RFRTM LST. In addition, days with less than 10% valid MERSI-LL LST values over the study area were excluded in the estimation of the SIFI to guarantee the reliability of the SIFI.

## 4. Results and Discussion

### 4.1. Retrieved Dawn–Dusk 1 km TIR LST

**Figure 4:**
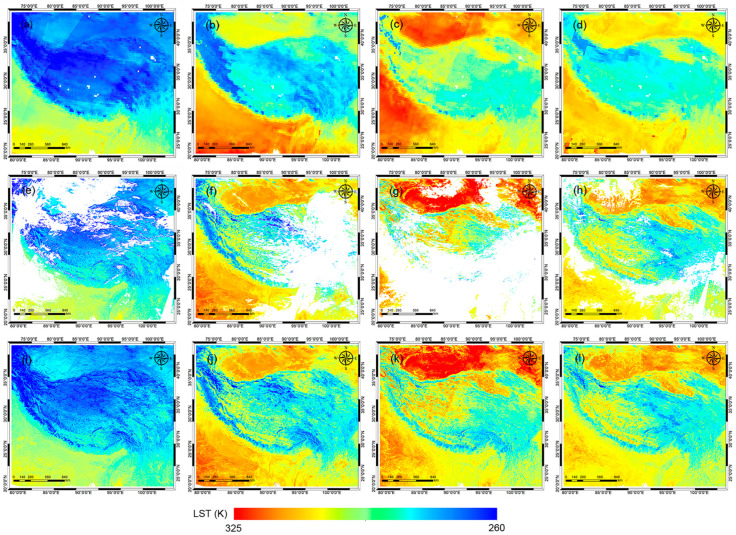
The 0.25° GLDAS LST (**a**–**d**), 1 km MERSI-LL LST (**e**–**h**), and 1 km RFRTM LST (**i**–**l**) at dusk on DOY 90 (**a**,**e**,**i**), DOY 180 (**b**,**f**,**j**), DOY 270 (**c**,**g**,**k**), and DOY 360 (**d**,**h**,**l**) over the target period.

Figure 4 shows the 0.25° GLDAS LST, 1 km FY-3E MERSI-LL LST, and 1 km RFRTM LST at dusk on DOY 90, DOY 180, DOY 270, and DOY 360 during the target period. Compared with the MERSI-LL LST, the GLDAS LST had a similar spatial pattern of LST on DOY 90 over most of the study area: low LST data (i.e., lower than 278 K) were mainly distributed in the north and on the edge of the Tibetan Plateau due to higher elevation, although the GLDAS LST was ~1.61 K lower in the area. On DOY 180, the phenomenon was different: the MERSI-LL LST had close values in the north and south of the Tibetan Plateau, while the GLDAS LST in the north of the plateau was evidentially lower than in the south of the plateau, where the GLDAS LST was ~2.52 K higher than the MERSI-LL LST. Results from the other two days were similar: the GLDAS LST was 1.23 K/0.95 K higher than the MERSI-LL LST in the south of the plateau and 2.74 K/4.45 K lower in the rest of the area on DOY 270/360. The discrepancies between the GLDAS LST and the MERSI-LL LST data resulted from uncertainties in imperfect land parameterizations and forcing fields, which may cause considerable drifts in the modelling of the GLDAS LST [62]. Moreover, because of the lower spatial resolution, the GLDAS LST, even though it was all-sky available, displayed far fewer details of the LST spatial patterns compared with the MERSI-LL LST.

In contrast, the RFRTM LST data not only exhibited a spatial pattern highly consistent with the MERSI-LL LST but also fully compensated for the missing MERSI-LL LST data. As shown in Table 3, over the target period, 52.86%/56.94% of dawn/dusk MERSI-LL LST data were available over the study area, meaning that half of the LST data were not valid due to cloud contamination. The dawn/dusk RFRTM LST was all-sky available, indicating that the RFRTM LST could fully compensate for the missing dawn/dusk LST data under cloudy conditions.

Additionally, blocking effects or mosaics were rarely observed in the RFRTM LST images, indicating the satisfactory performance of the method with respect to acquiring the fine-resolution LST from the coarse-resolution reanalysis data.

**Figure 5:**
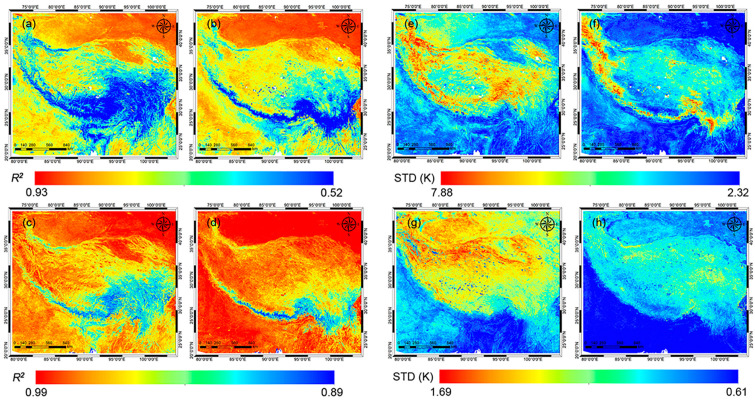
Spatial patterns of evaluation indices when compared with the original MERSI-LL LST over the target period: *R*^2^ of 0.25° GLDAS LST (**a**,**b**), *R*^2^ of 1 km RFRTM LST (**c**,**d**), STD of 0.25° GLDAS LST (**e**,**f**), and STD of 1 km RFRTM LST (**g**,**h**). (**a**,**c**,**e**,**g**) are dusk cases, and the rest are dawn cases.

Spatial patterns of evaluation metrics of the GLDAS LST and the RFRTM LST including *R*^2^ and STD when compared with the MERSI-LL LST are shown in Figure 5. Compared with the GLDAS LST, the *R*^2^ of the RFRTM LST data ranged from 0.89 to 0.99, which was 0.06 to 0.36 higher; meanwhile, the STD ranged from 0.61 K to 1.69 K, which was 1.71 to 1.19 K lower, indicating better agreement with the MERSI-LL LST over the period. Moreover, the better agreement between the dawn RFRTM LST and MERSI-LL LST compared with the dusk results could be attributed to the following reason: the variation in LST descriptors from GLDAS, which were temporally interpolated to the MERSI-LL observation time, was smaller at dawn than dusk, thus inducing lower uncertainty in the RFRTM LST. In addition, over the areas outside of the Tibetan Plateau, the RFRTM LST data also showed better agreement with the MERSI-LL LST than over the plateau with a smaller STD, which could result from (1) higher agreement between the GLDAS LST and the MERSI-LL LST over these areas and (2) lower spatial heterogeneity, which could lead to higher uncertainty in the trained model.

### 4.2. Intercomparison with Satellite LST

#### 4.2.1. Intercomparison of Retrieved TIR LST and Official TIR LST

**Figure 6:**
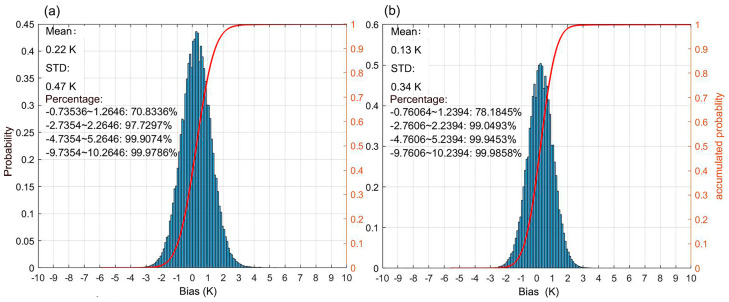
Histograms of bias in retrieved dawn (**a**) and dusk (**b**) MERSI-LL LST compared with the official MERSI-LL LST. The sample size was 1.15 × 10^9^ and 1.22 × 10^9^ at dawn and dusk, respectively.

Figure 6 shows histograms of bias in retrieved dawn and dusk FY-3E MERSI-LL LST data compared with official MERSI-LL LST data from CMA (http://satellite.nsmc.org.cn/, accessed on 1 November 2024)) over the target period. The mean bias of 0.22 K and 0.13 K at dawn and dusk revealed a negligible systematic difference between the two LST datasets. The 0.34–0.47 K STD and coefficients of 0.99 further demonstrated that the retrieved LST had a satisfactory accuracy compared with the official LST products, confirming that the TIR retrieval method in Section 3.1 was reliable over the study area.

#### 4.2.2. Intercomparison of RFRTM LST and Original TIR LST

**Figure 7:**
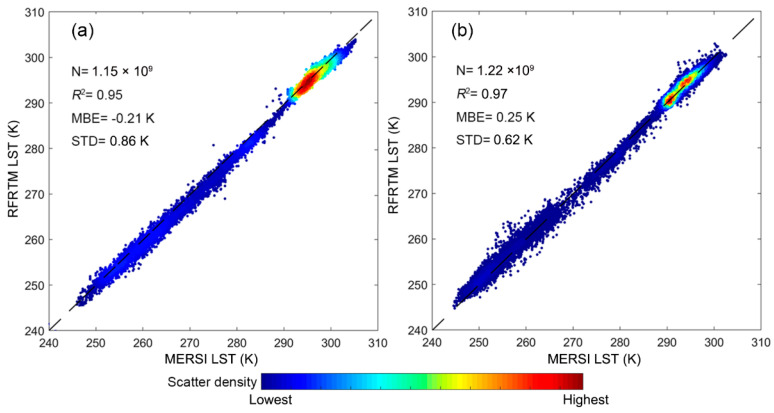
Scatter plots between the 1 km clear-sky RFRTM LST and the corresponding MERSI-LL LST over the study area during the target period at dusk (**a**) and dawn (**b**). N denotes the sample size.

Figure 7 shows scatter plots between the RFRTM LST and the MERSI-LL LST over the target period. The dusk/dawn RFRTM LST yielded systematic bias with an MBE of −0.21 K/0.25 K when compared with the original MERSI-LL LST. The STD from 0.86 K to 0.62 K indicated that the two LSTs highly agreed with each other, thus demonstrating the robustness of the constructed RFRTM model. The MBE between the RFRTM LST and the MERSI-LL LST was ignorable, although the observation of the 0.25° GLDAS had systematic error against the corresponding observations at 1 km scale. This result indicated that the RFRTM model effectively eliminated the bias in the reanalysis data from the remote sensing data, as demonstrated by Equations (5) and (6).

#### 4.2.3. Intercomparison of RFRTM LST and TIR–PMW Merged All-Sky LST

**Figure 8:**
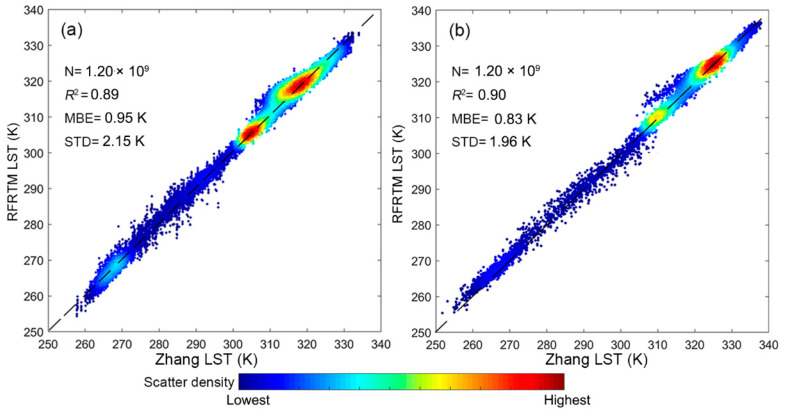
Scatter plots between the 1 km all-sky RFRTM LST and the corresponding Zhang LST over the study area during the target period at dusk (**a**) and dawn (**b**). N denotes the sample size.

Figure 8 shows the scatter plots between the all-sky RFRTM LST and the Zhang LST: the MBE was 0.95 K/0.83 K at dawn/dusk, revealing non-negligible systematic bias; the corresponding STD was 2.15 K/1.96 K, indicating a larger difference between the two 1 km all-sky LSTs compared with the RFRTM LST and MERSI-LL LST.

**Figure 9:**
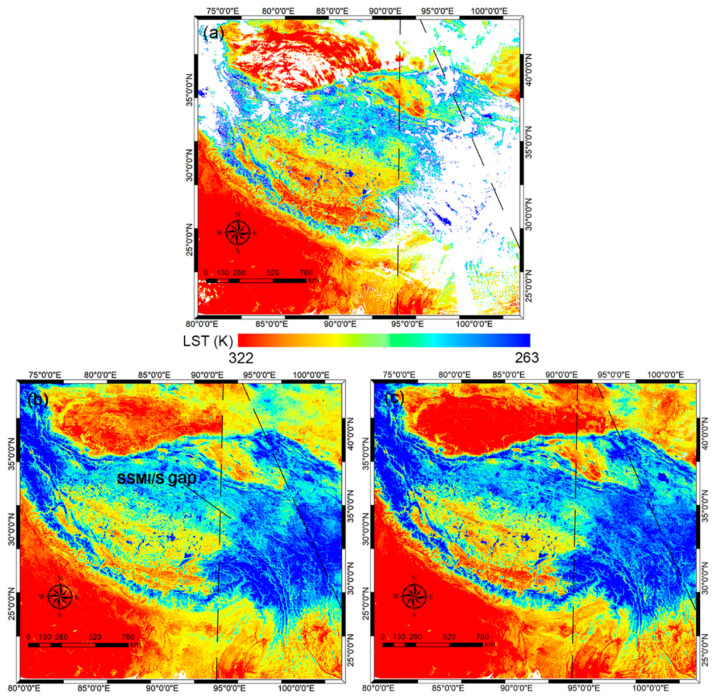
The dusk 1 km MERSI-LL LST (**a**), Zhang LST (**b**), and RFRTM LST (**c**) on 30 June 2024.

To investigate the difference, Figure 9 presents the dusk 1 km MERSI-LL LST, Zhang LST, and RFRTM LST on 30 June 2024 as an example. Due to cloud contamination, nearly half of the pixels were missing in the MERSI-LL LST. Although two all-sky LSTs filled in the missing LST values over the cloudy-sky area, some noticeable discrepancies existed between their images; over most of the area, both the RFRTM LST and the Zhang LST exhibited a similar spatial pattern to the MERS LST. However, the latter showed an underestimation over barren land areas, such as the Gobi desert, due to larger inconsistency between the PMW LST and the TIR LST. The inconsistency arose from the PMW thermal sampling depth, which could introduce a subsurface temperature to the retrieved surface skin temperature [82,83]. Moreover, there was clear spatial discontinuity at the boundary between the SSMI/S swath gap-covered area and the remaining area, which could arise from the uncertainty induced by the gap-filling algorithm used for PMW BT reconstruction in Zhang’s method. In contrast, the RFRTM LST barely suffered from the blocking effect and displayed better spatial continuity, benefiting from the satisfactory downscaling ability of the RF mapping transform. In addition, avoiding the thermal sampling depth effect and data unavailability effect over gap-covered area data from SSMI/S observations, the RFRTM LST yielded no evident bias over all land cover types or SSMI/S swath gap-filled areas, thus improving image quality. The results were quite similar for other days.

**Figure 10:**
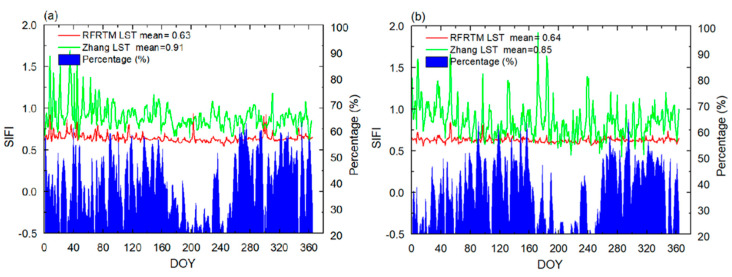
SIFI variation in the RFRTM LST and the Zhang LST over the target period. The blue area at the bottom of the figure denotes the daily percentage of valid MERSI-LL LST pixels in the study area. (**a**,**b**) The dusk and dawn time conditions, respectively.

The above result was supported by the comparison of the SIFI between the Zhang LST and the RFRTM LST, as shown in Figure 10. The mean SIFI of the Zhang LST was 0.91/0.85. In contrast, the RFRTM LST had a lower mean SIFI, demonstrating that the RFRTM LST image was capable of maintaining more consistent spatial patterns with the MERSI-LL LST and also showed better image quality. In addition, an opposite trend was observed between the SIFI and the sample size of the MERSI-LL LST at the intra-annual scale. This indicated that the MERSI-LL LST sample size positively affected the image quality of the two all-sky LSTs since more valid MERSI-LL LSTs contributed to a stable model structure and better robustness and consequently led to less bias on their LST images. However, the SIFI of the RFRTM LST was less sensitive to the number of MERSI-LL LST data, which could be attributed to less dependency on sample size for training a stable RF regression mapping transform in the merging of MERSI-LL-GLDAS data than MERSI-LL-SSMI/S data.

### 4.3. Validation of RFRTM LST Against Ground Measurements

**Figure 11:**
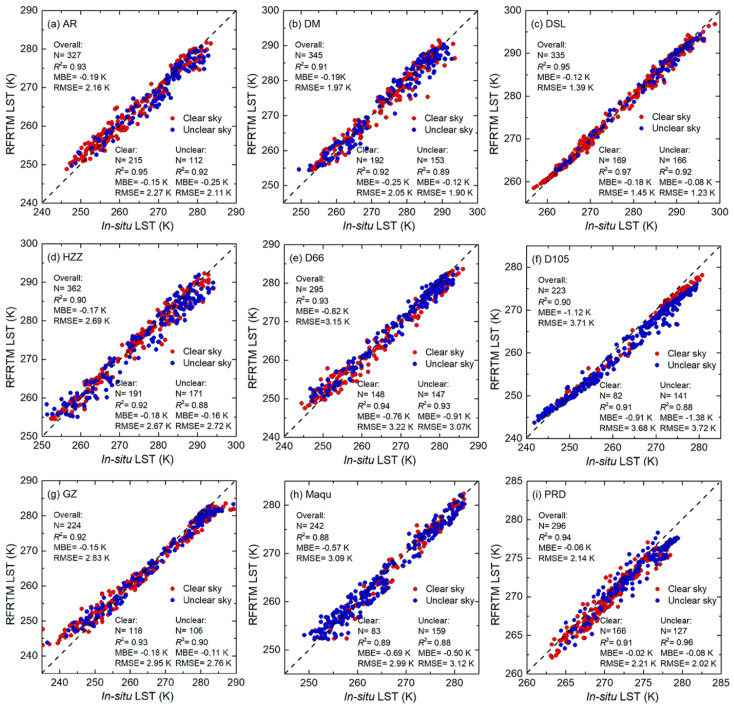
Scatter plots between the dusk RFRTM LST and in situ LST of ground sites over the Heihe River Basin (**a**–**d**) and the Tibetan Plateau (**e**–**i**).

Figure 11 shows scatter plots of dusk RFRTM LST against in situ LST. Total validation results of the RFRTM LST, Zhang LST, and MERSI-LL LST are also listed in Table 4, Table 5 and Table 6. Considering the difference between the climate and topography of the Tibetan Plateau and the Heihe River Basin, the validation results over the two areas were analyzed separately (see Table 4 and Table 5 for the Tibetan Plateau and Table 6 for the Heihe River Basin). For the purpose of analysis, as given in Table 7, the thermal spatial heterogeneity of the 1 km site-located pixel was calculated using the STD of the 250 m MERSI-LL LST, which could be retrieved from its 250 m L1 radiance product following the steps outlined in Section 3.3 and located in the 1 km pixel. The ground-measured average soil moisture (at a 2 cm depth) over the target period at all sites is also given in Table 7.

(1) Tibetan Plateau

Taking the dusk results as an example, among all the sites, the accuracies of the MERSI-LL LST and RFRTM LST were similar with negligible differences of no more than 0.10 K/0.14 K considering the MBE/RMSE, indicating that the trained RFRTM model maintained good robustness when generalized over the next period. The accuracy of the RFRTM LST was the highest at PRD with an MBE of −0.06 K and an RMSE of 2.14 K and the lowest at D105 with an MBE of −1.12 K and an RMSE of 3.71 K. It was obvious that the accuracy had a negative correlation with the thermal spatial heterogeneity: in PRD, the spatial heterogeneity was 0.22 K, which was 0.53 K/0.75 K/0.22 K/0.40 K lower than D66/D105/GZ/MQ, respectively, while the RMSE was 0.99/0.55 K/0.97 K/0.93 K higher. This phenomenon could be attributed to the soil moisture: higher soil moisture led to lower thermal spatial heterogeneity inside the MERSI-LL 1 km pixel, leading to reduced uncertainty, which arose from scale mismatching between the in situ site’s FOV and the 1 km pixel during validation. In addition, no evident difference was shown in the accuracy of the clear-sky and cloudy-sky RFRTM LST, demonstrating that the constructed RFRTM trained under clear-sky could be satisfactorily generalized under all-sky conditions.

Similar to the RFRTM LST, the Zhang LST also had the highest accuracy at PRD with an MBE of −0.12 K and an RMSE of 2.07 K and lowest accuracy at D105 with an MBE of −1.27 K and an RMSE of 4.22 K. However, compared with the RFRTM LST, the Zhang LST had a more obvious underestimation of the in situ LST, with an MBE of −0.12 K at PRD, −0.66 K at MQ, −1.22 K at GZ, −1.27 K at D105, and −0.84 K at D66. The results could be explained by the underestimation of the PMW LST suffering from the PMW thermal sampling depth effect. For example, the RMSE at D105 was 0.88 K and 0.62 K lower than those at GZ and MQ because the soil moisture in D105 was only about half of that in D66 and MQ, which implied a larger PMW thermal sampling depth at the site [84]. In addition, the underestimation was more significant at sites with lower soil moisture, further indicating that the PMW thermal sampling depth effect played a more vital role in determining the accuracy of the Zhang LST. The results were similar at dawn, while all 1 km LST data were in close agreement with the in situ LST data due to thermal lower spatial thermal heterogeneity compared with the results at dusk.

(2) Heihe River Basin

Similarly, taking dusk as an example, over all the sites, the RFRTM LST was also highly consistent with the MERSI-LL LST with slight differences. The RFRTM LST had the highest accuracy with an MBE of −0.17 K and an RMSE of 1.51 K at the DSL site, with the lowest accuracy observed at HZZ with an MBE of −1.01 K and an RMSE of 2.97 K. At AR and DM, the RFRTM LST had similar accuracy with an MBE of approximately −0.20 K and an RMSE of 2.16 K. The accuracy of the RFRTM LST was found to be negatively related to the soil moisture, which was an indicator of surface thermal heterogeneity: the soil moisture at DSL was 21.25%, which was the highest, while the moisture at HZZ was 6.69% and lower than that observed at other sites. This result indicated that the discrepancy in the accuracy of the RFRTM LST between sites mainly depended on the surface thermal heterogeneity of the sites. The RFRTM LST also demonstrated consistent accuracy under both clear-sky and cloudy-sky conditions.

The Zhang LST also had the highest accuracy at the DSL site. The RMSE was 1.37 K and was closest to the RMSE of the RFRTM LST among all sites due to the following reason: on one hand, the soil moisture at this site was two to three times higher than that at other sites due to higher water capacity of the swamp meadow [85], thus significantly weakening the PMW thermal sampling depth effect on the estimated LST at the site; on the other hand, the highest soil moisture may also indicate the lowest thermal spatial heterogeneity among these sites. This also explained why the RMSE was highest at the HZZ site with desert land cover, over which the soil moisture was the lowest and the PMW thermal sampling effect may be the most significant. However, similar to the results over the Tibetan Plateau, the Zhang LST also yielded significant underestimation against in situ LST over all sites due to the PMW thermal sampling depth effect with MBEs from −0.28 K at DSL to −1.56 K at HZZ.

In conclusion, regarding the validation results, the RFRTM LST had similar accuracy to the MERSI-LL LST, indicating the robustness of the model. Moreover, it also had similar performance between clear- and cloudy-sky conditions, revealing the satisfactory generalized ability of the trained RFRTM model under all-sky conditions. Furthermore, compared with the MERSI-LL-SSMI/S merged all-sky LST, the RFRTM LST had better accuracy because it could avoid the issue of PMW thermal sampling depth, which could lead to underestimation of the TIR–PMW merged LST.

### 4.4. Performance over the PMW Swath Gap-Covered Area

**Figure 12:**
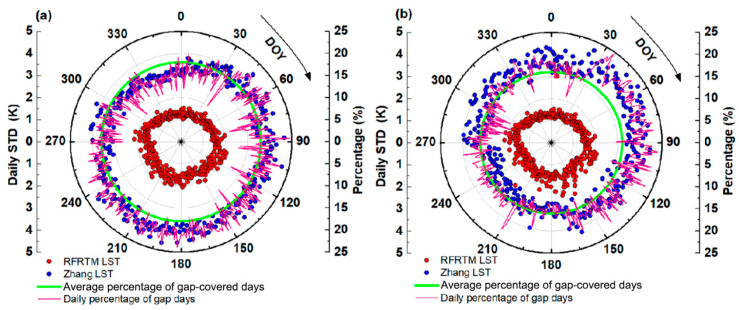
STD of the RFRTM LST (red circle) and the Zhang LST (blue circle) over the SSMI/S swath gap-covered area when compared with the MERSI-LL LST over the target period. The daily percentage (pink line) and averaged percentage (green line) of the gap-covered MERSI-LL pixel number over the entire study area are also shown. (**a**) and (**b**) The dusk and dawn conditions, respectively.

Located in the low–mid-latitude area, the study area frequently suffered from the polar-orbit SSMI/S gap between the adjacent swath. Figure 12 shows the STD of the RFRTM LST and the Zhang LST over the SSMI/S swath gap-covered area when compared with the MERSI-LL LST over the target period. As shown in this figure, during the target period, 11–24% of the study area was covered with the SSMI/S swath gap on a specific day. Despite the Zhang LST being processed with a gap-filling algorithm, the intercomparison results with the MERSI-LL LST shown in Figure 9 revealed the non-negligible bias in the Zhang LST over the swath gap-filled area. To further investigate the issue, the Zhang LST was compared with the RFRTM LST over the swath gap-covered area. As shown in Figure 12, it was evident that the agreement between the Zhang LST and the MERSI-LL LST was negatively correlated with the percentage of gap-covered pixels. In contrast, avoiding the gap issue, the RFRTM LST results were consistent with those of the original MERSI-LL LST.

**Figure 13:**
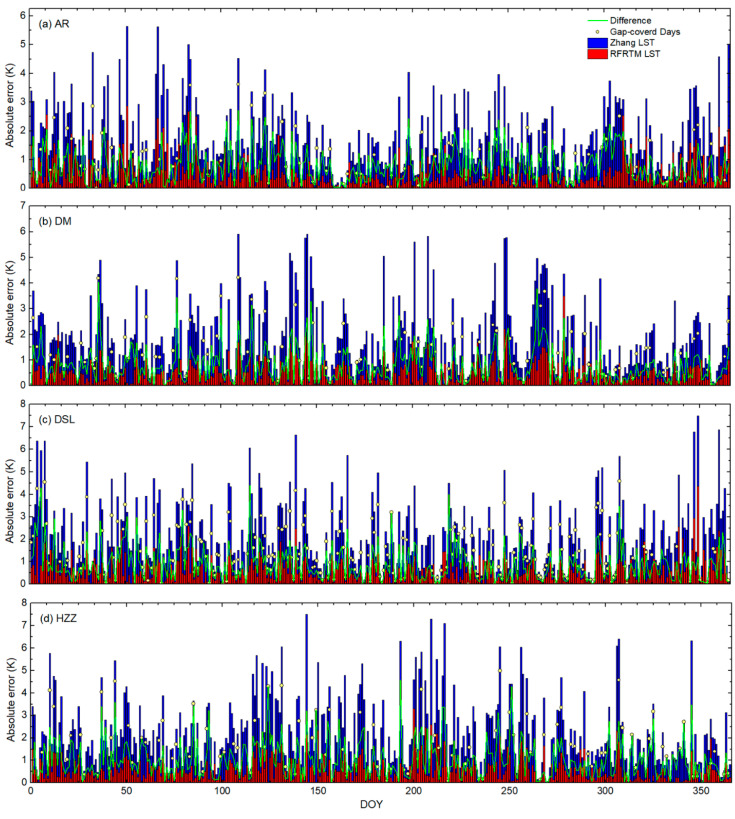
Daily absolute error (denoted by length of the bar) of the dusk RFRTM LST and the Zhang LST against the in situ LST at AR, DM, DSL, and HZZ during the target period.

A similar phenomenon was also found in the validation of the two all-sky LSTs against the ground measurements. The daily absolute error of the dusk RFRTM LST and Zhang LST against the in situ LST at AR, DM, DSL, and HZZ during the target period is illustrated as an example in Figure 13. Across all of the ground sites, the RMSE values of the Zhang LST appeared to be higher when covered with the swath gap compared with other times. When the sites were inside the swath gap, the average absolute error increased by 1.22 K at the AR site, by 1.05 K at the DM site, by 1.27 K at the DSL site, and by 1.82 K at the HZZ site. Therefore, the accuracy difference between the two LSTs was apparently larger on the swath-gap-covered days. In contrast, benefiting from the good spatiotemporal continuity of the GLDAS data, the absolute error of the RFRTM LST barely showed a difference between gap-covered and no-gap cases: the maximum (minimum) average absolute error absolute difference was 0.34 K (0.14 K) at the HZZ site (AR) site. The results for the Tibetan Plateau were similar and, therefore, are not elaborated here.

### 4.5. Variation in Method Performance with Main Land Covers and Models

#### 4.5.1. Main Land Covers

In order to investigate the difference in the method performance between main land covers, the original land covers from MCD12Q (waterbodies are excluded) of each 1 km pixel were reclassified into the following three classes: dense vegetation, sparse vegetation, and barren land [36]. The reclassification scheme and the corresponding area proportion are provided in Table 8, with the reclassified land cover type hereafter being termed “static land cover”. However, if the dynamic change in land cover is considered, the land cover type of each 1 km pixel can be reclassified into the same three classes according to [86]: (1) dense vegetation area (NDVI > 0.5), (2) sparse vegetation area (0.2 ≤ NDVI ≤ 0.5), and (3) barren land area (NDVI < 0.2). The reclassified land cover type considering the dynamic change is hereafter termed “dynamic land cover.” Possible different land cover after considering dynamic change is given in Table 8, and the area proportion of dynamic land covers is given in Table 9.

**Figure 14:**
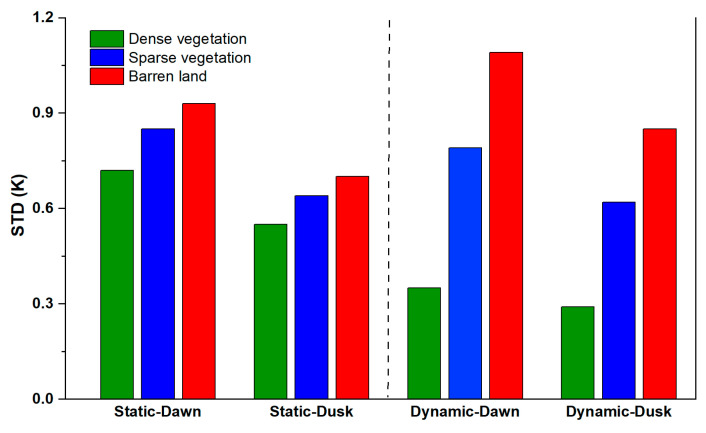
STD of the RFRTM LST compared with the MERSI-LL LST during the target period over the dense vegetation area, sparse vegetation area, and barren land area based on static land covers and dynamic land covers.

Figure 14 shows the STD of the RFRTM LST compared with the MERSI-LL LST during the target period over the dense vegetation area, sparse vegetation area, and barren land area based on static land covers and dynamic land covers. Taking the results at dawn as an example, among static land covers, the STD was highest in the barren land area, attributed to the following two main reasons: (1) the NDVI over the barren land area was relatively smaller than that of the other two land covers, which weakened the effects of the NDVI in enhancing the robustness of the RFRTM model, and (2) the specific heat capacity of barren land was generally lower, which could lead to larger variation in some reanalysis variables (e.g., soil temperature) on a temporal scale, thus leading to higher uncertainty of the interpolated GLDAS LST descriptors used for estimating the RFRTM LST over this land cover. Similarly, when compared with the dense vegetation area, the sparse vegetation area had a lower specific heat capacity. Moreover, as shown in Figure 2, sparse vegetation was generally located at higher altitudes than dense vegetation. These two factors led to higher STD of the sparse vegetation area than the dense vegetation area. After considering the dynamic change in the land cover, the conclusion still held, whereas the STD difference between the three land covers was obviously larger compared with the conditions under the static land cover. The main reason was that the majority of the sparse vegetation area was reclassified to either barren land area or dense vegetation area, as outlined in Table 8 and Table 9. In other words, without considering actual dynamic land cover change, the static dense vegetation (barren land) area included the barren land (dense vegetation) conditions, resulting in a larger (smaller) STD.

#### 4.5.2. Models

**Figure 15:**
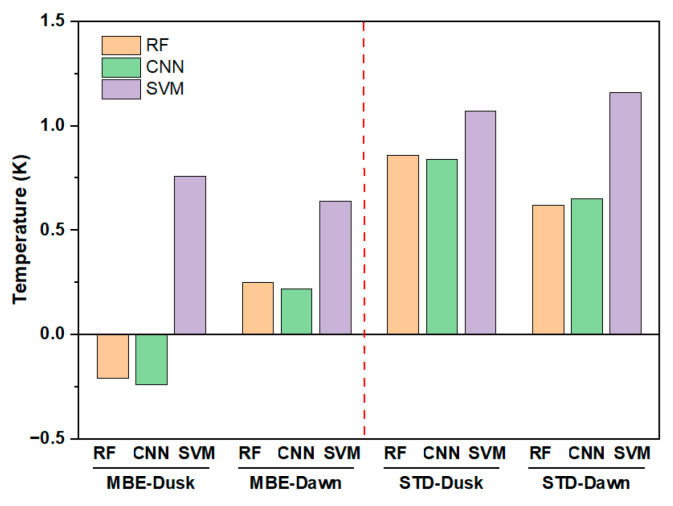
Comparison with the MERSI-L LST over the target period. The MBE and STD of the RFRTM, CNNRTM, and SVMRTM LSTs.

In our preliminary experiment, two widely used machine learning models—convolutional neural networks (CNNs) and the support vector machine (SVM)—were selected to replace the RF model in the method and estimate the same dawn–dusk LST (hereafter called the CNNRTM LST and SVMRTM LST). Figure 15 shows the MBE and STD of the RFRTM, CNNRTM, and SVMRTM LSTs when intercompared with the MERSI-L LST during the target period. Table 10 shows the *R*^2^ of the three types of LST when intercompared with the MERSI-L LST and also gives the computing time for implementing the corresponding model.

Among the three LSTs, the SVMRTM LST showed the lowest accuracy, with obvious bias and larger STD compared with the MERSI-LL LST. The difference in the MBE and STD between the RFRTM LST and CNNRTM LST was slight (less than 0.04 K). Table 11 further lists the maximum difference in the *R*^2^, MBE, and RMSE between the RFRTM LST-CNNRTM LST over the ground stations during the target period. The result also confirmed the negligible difference in terms of the accuracy between the two LSTs. However, when considering the computing time, it was found that the time cost of implementing the CNNRTM method was 50% more than that observed when using the RFRTM method with the same computing power. Therefore, given comprehensive consideration of the accuracy and computing efficiency, the RF was used as the primary model in this study.

### 4.6. Limitations and Implications

#### 4.6.1. Limitations

Although the RFRTM method proposed in this study contributes to filling the gap in the 1 km all-sky LST at dawn–dusk time, the limitations of this method should also be acknowledged. First, to the best of our knowledge, FY-3E MERSI-LL is currently the only public sensor that can provide dawn–dusk 1 km TIR LST observations. Therefore, unfortunately, it is not feasible to use other satellite TIR data as an alternative to meet the objective of this study, meaning that the method currently relies on MERSI-LL data. Second, this study used the MERSI-LL TIR radiance product to retrieve LST data instead of directly using the official daily 1 km LST data as the LST product had only been available to the public from 2023, which was not sufficient for training the RFRTM model. However, the complex retrieval process weakened the method’s portability. Fortunately, with the continuing accumulation of the MERSI-LL official TIR LST online, it was practical to directly utilize the official MERISI LST for implementing the method. Third, due to the limited computing power, the method was only applied to GLDAS data over the Tibetan Plateau and the surrounding area. In the mechanism, the method was processed pixel by pixel based on a machine learning model, and it was self-adaptive under various cases (i.e., different areas and reanalysis data sources such as CLDAS, MERRA, and ERA-5). Nevertheless, further experiments are still necessary to evaluate the general ability of the method using other region and reanalysis data.

#### 4.6.2. Implications

This study proposed the RFRTM method for generating dawn–dusk 1 km all-sky LST data by merging TIR and reanalysis data, a topic that has not been investigated in previous studies. The core component of the method was the RF mapping transform, which was constructed between the time series of the LST and its multiple descriptors from the reanalysis data in the temporal dimension. The good performance of the RFRTM LST when validated with ground measurements under all-sky conditions demonstrated the capability of the RF mapping transform to directly derive a reliable all-sky high-spatial-resolution LST from a low-resolution LST provided that the coarse-resolution data had multiple LST descriptor time series with sufficient samples. The results may reveal that similar methods can be applied to derive other all-sky high-resolution geophysical parameters through merging TIR and reanalysis data such as albedo, LAI, NDVI, and so on, which are also unavailable under cloudy-sky conditions, facilitating related land process models such as the hydrologic cycle, carbon cycle, radiation budget, and so on.

In addition, considering that the trained RFRTM method from the training period had good performance when applied to the subsequent target period, a real-time/near-real-time dawn–dusk all-sky 1 km LST dataset can be derived from MERSI-LL-GLDAS datasets based on a model trained from the last period within an appropriate time window. Combined with current day–night 1 km all-sky LST datasets, a nearly 6 h (i.e., four times daily) 1 km LST dataset is expected to be generated, which would be highly valuable for associated studies and applications in the future.

## 5. Conclusions

Merging TIR–PMW data and merging TIR–reanalysis data are two mainstream approaches to derive 1 km all-sky LST, which has become more urgently required in recent years. However, current TIR–PMW and TIR–reanalysis merged 1 km all-sky LST products can only provide data from the observation times of MODIS, which has been the sole TIR sensor used in previous merging studies. Therefore, there is still a lack of 1 km all-sky data at other times, such as dawn–dusk. In order to fill the gap in the 1 km all-sky remotely sensed LST data during dawn–dusk, through merging more than 2 years of 1 km TIR observation data from the young dawn–dusk-orbit sensor FY-3E MERSI-LL and GLDAS using a random forest model, this study proposed an RFRTM method to estimate dawn–dusk 1 km all-sky LST. The trained RFRTM model was demonstrated to have satisfactory robustness, indicated by a 0.62–0.86 K STD of the estimated clear-sky LST compared with the original MERSI-LL LST over the Tibetan Plateau and its surrounding area. Moreover, the RFRTM indicated acceptable accuracy when validated against ground-measured LST under all-sky conditions. In addition, the RFRTM LST outperformed the 1 km dawn–dusk LST merged from MERSI-LL-SSMI/S (i.e., TIR–PMW) with higher accuracy and better image quality over the PMW gap-covered area. Therefore, the RFRTM is practical for estimating a reliable 1 km dawn–dusk all-sky LST, helping to fill the gap in current 1 km all-sky LST products for dawn–dusk times. Therefore, based on this study, a long-term 6 h 1 km all-sky LST dataset is expected to be produced, coupled with current day–night 1 km LST products, which would be promising for future applications at regional and global scales.

## Figures and Tables

**Table 1:** Information on the measurements at ground sites.

Site	Location (°E, °N)	Altitude (m)	Land Cover	Instrument and Height (m)	Diameter of FOV (m)	Data Interval (min)
D66	93.78, 35.32	4585	Alpine grassland	CNR1, 2.43	18.14	10
D105	91.94, 33.06	5039	Alpine grassland	CNR1, 1.34	10.00	10
GZ	84.06, 32.31	4394	Barren land	CNR1, 1.49	11.12	10
MQ	102.14, 33.89	3433	Alpine plain grass	CNR1, 1.50	11.20	10
PRD	86.81, 27.96	5035	Alpine plain grass	CNR1, 2.00	14.93	10
AR	100.41, 37.98	3517	Alpine grassland	CNR4, 6.00	44.78	10
DM	100.37, 38.86	1536	Cropland	CNR4, 12.00	89.57	10
DSL	98.94, 38.84	3788	Swamp meadow	CNR4, 6.00	44.78	10
HZZ	100.32, 38.77	1732	Desert	CNR4, 2.50	18.66	10

**Table 2:** Selected LST descriptors.

Basic Descriptors	Ancillary Descriptors
Net longwave radiation; longwave downward flux; soil moisture profile (0–10 cm depth); canopy surface water; snow depth water equivalent; NDVI	Soil temperature profile (surface, 0–10 cm depth); wind speed; air temperature; albedo

**Table 3:** Percentage of 1 km pixels with valid MERSI-LL LST and RFTRTM LST over the study area during the target period.

Condition	MERSI-LL LST	RFTRTM LST (MERSI-LL + GLDAS)
Dawn	52.86%	100%
Dusk	56.94%	100%

**Table 4:** Validation of the dusk RFRTM LST, the Zhang LST, and the original MERSI-LL LST against in situ LST over the target period over the Tibetan Plateau. N denotes the sample size.

Case	Condition	N	RFRTM LST			Zhang LST			MERSI-LL LST		
			MBE (K)	RMSE (K)	*R* ^2^	MBE (K)	RMSE (K)	*R* ^2^	MBE (K)	RMSE (K)	*R* ^2^
D66	All conditions	295	−0.83	3.15	0.93	−0.85	3.12	0.92			
	Clear sky	148	−0.76	3.22	0.94	−0.92	3.15	0.91	−0.69	3.20	0.94
	Unclear sky	147	−0.91	3.07	0.93	−0.77	3.09	0.92			
D105	All conditions	223	−1.21	3.71	0.89	−1.37	4.22	0.86			
	Clear sky	82	−0.91	3.68	0.91	−1.06	4.28	0.88	−0.81	3.57	0.90
	Unclear sky	141	−1.38	3.72	0.88	−1.55	4.19	0.85			
GZ	All conditions	224	−0.15	2.83	0.92	−1.20	3.53	0.90			
	Clear sky	118	−0.18	2.95	0.93	−1.28	3.52	0.91	−0.13	2.81	0.93
	Unclear sky	106	−0.11	2.76	0.90	−1.12	3.64	0.89			
MQ	All conditions	242	−0.57	3.09	0.88	−0.67	3.16	0.89			
	Clear sky	83	−0.69	2.99	0.89	−0.74	3.12	0.91	−0.62	2.96	0.90
	Unclear sky	159	−0.50	3.12	0.88	−0.63	3.19	0.88			
PRD	All conditions	184	−0.05	2.16	0.93	−0.14	2.24	0.93			
	Clear sky	105	−0.02	2.21	0.91	−0.18	2.26	0.92	−0.02	2.24	0.93
	Unclear sky	79	−0.08	2.02	0.96	−0.09	2.20	0.94			

**Table 5:** Validation of the dawn RFRTM LST, the Zhang LST, and the original MERSI-LL LST against in situ LST during the target period over the Tibetan Plateau. N denotes the sample size.

Case	Condition	N	RFRTM LST			Zhang LST			MERSI-LL LST		
			MBE (K)	RMSE (K)	*R* ^2^	MBE (K)	RMSE (K)	*R* ^2^	MBE (K)	RMSE (K)	*R* ^2^
D66	All conditions	278	−0.44	2.88	0.91	−0.62	2.92	0.91			
	Clear sky	132	−0.36	2.92	0.90	−0.67	2.95	0.91	−0.31	2.86	0.92
	Unclear sky	146	−0.51	2.87	0.91	−0.57	2.89	0.91			
D105	All conditions	243	−0.59	3.48	0.92	−1.27	4.13	0.86			
	Clear sky	109	−0.61	3.58	0.91	−1.01	4.02	0.87	−0.54	3.42	0.91
	Unclear sky	134	−0.58	3.42	0.94	−1.05	4.19	0.86			
GZ	All conditions	251	−0.12	2.25	0.92	−0.88	3.28	0.92			
	Clear sky	123	−0.14	2.23	0.93	−0.94	3.22	0.92	−0.15	2.31	0.93
	Unclear sky	128	−0.11	2.26	0.91	−0.82	3.32	0.90			
MQ	All conditions	233	−0.39	2.85	0.92	−0.55	3.00	0.89			
	Clear sky	112	−0.35	2.73	0.90	−0.57	3.01	0.90	−0.32	2.66	0.92
	Unclear sky	121	−0.42	2.92	0.92	−0.53	2.99	0.89			
PRD	All conditions	176	0.14	1.68	0.94	−0.30	1.93	0.94			
	Clear sky	92	0.12	1.71	0.93	−0.22	1.96	0.95	0.12	1.77	0.94
	Unclear sky	84	0.17	1.62	0.95	−0.39	1.90	0.92			

**Table 6:** Validation of the RFRTM LST, the Zhang LST, and the original MERSI-LL LST against in situ LST during the target period over the Heihe River Basin. N denotes the sample size.

Case	Condition	Time	N	RFRTM LST			Zhang LST			MERSI-LL LST		
				MBE (K)	RMSE (K)	*R* ^2^	MBE (K)	RMSE (K)	*R* ^2^	MBE (K)	RMSE (K)	*R* ^2^
AR	All conditions	Dusk	327	−0.18	2.16	0.95	−0.73	2.56	0.90			
		Dawn	354	−0.12	2.01	0.94	−0.43	2.32	0.91			
	Clear sky	Dusk	215	−0.15	2.27	0.92	−0.74	2.59	0.90	−0.11	2.19	0.91
		Dawn	215	−0.17	1.97	0.93	−0.41	2.42	0.90	−0.12	1.81	0.93
	Unclear sky	Dusk	112	−0.25	2.11	0.93	−0.71	2.50	0.91			
		Dawn	139	−0.05	2.04	0.94	−0.45	2.27	0.91			
DM	All conditions	Dusk	345	−0.19	1.97	0.91	−0.58	2.38	0.92			
		Dawn	351	0.12	1.32	0.93	−0.51	2.05	0.89			
	Clear sky	Dusk	192	−0.25	2.05	0.92	−0.56	2.45	0.90	−0.28	2.02	0.92
		Dawn	220	0.11	1.24	0.92	−0.51	2.11	0.90	0.02	2.12	0.93
	Unclear sky	Dusk	153	−0.12	1.90	0.89	−0.60	2.31	0.91			
		Dawn	131	0.14	1.36	0.94	−0.52	1.97	0.89			
DSL	All conditions	Dusk	335	−0.13	1.34	0.95	−0.29	1.57	0.93			
		Dawn	328	0.04	1.16	0.93	−0.21	1.37	0.91			
	Clear sky	Dusk	169	−0.08	1.45	0.97	−0.33	1.51	0.92	−0.05	1.35	0.98
		Dawn	178	−0.02	1.18	0.94	−0.21	1.32	0.91	−0.10	0.89	0.95
	Unclear sky	Dusk	166	−0.18	1.23	0.92	−0.24	1.63	0.93			
		Dawn	150	0.11	1.14	0.93	−0.21	1.45	0.91			
HZZ	All conditions	Dusk	362	−0.17	2.69	0.90	−1.56	3.92	0.85			
		Dawn	362	−0.03	2.54	0.91	−1.00	3.33	0.84			
	Clear sky	Dusk	191	−0.18	2.66	0.92	−1.52	3.89	0.85	−0.12	2.59	0.92
		Dawn	211	−0.09	2.55	0.91	−0.92	3.25	0.83	−0.11	2.45	0.92
	Unclear sky	Dusk	171	−0.16	2.72	0.88	−1.59	3.95	0.84			
		Dawn	151	0.05	2.53	0.92	−1.12	3.45	0.84			

**Table 7:** Averaged thermal spatial heterogeneity and soil moisture (2 cm depth) of ground sites.

Site	Time	Thermal Spatial Heterogeneity (K)	Soil Moisture (%)
AR	Dusk	0.42	13.3
	Dawn	0.32	15.6
DM	Dusk	0.35	15.1
	Dawn	0.31	18.9
DSL	Dusk	0.12	29.1
	Dawn	0.13	31.2
HZZ	Dusk	0.64	9.8
	Dawn	0.48	10.7
D66	Dusk	0.75	12.4
	Dawn	0.57	14.7
D105	Dusk	0.97	9.2
	Dawn	0.71	11.1
GZ	Dusk	0.44	19.6
	Dawn	0.32	22.9
MQ	Dusk	0.62	16.6
	Dawn	0.43	18.2
PRD	Dusk	0.22	27.2
	Dawn	0.19	34.7

**Table 8:** The original land cover from MCD12Q and its corresponding area proportion, static land cover, and different possible land cover after considering dynamic change over the study area. It should be noted that land covers without dynamic change (i.e., permanent snow and ice) are not listed.

Land Cover from MCD12Q1	Area Proportion	Static Land Cover	Possible Different Land Cover After Dynamic Change
Deciduous needleleaf forest	5.24%	Dense vegetation	Sparse vegetation/barren land
Deciduous broadleaf forest	0.25%		
Mixed forest	1.17%		
Closed shrubland	0.14%	Sparse vegetation	Dense vegetation/barren land
Open shrubland	0.31%		
Woody savanna	0.72%		
Savanna	36.52%		
Grassland	3.23%		
Cropland	18.56%		
Cropland–natural vegetation	1.02%		
Permanent wetland	1.34%		
Barren land	30.89%	Barren land	Dense vegetation/sparse vegetation

**Table 9:** Land cover after considering dynamic change and its corresponding area proportion over the study area.

Land Cover After Considering Dynamic Change	Area Proportion
Dense vegetation	21.57%
Sparse vegetation	25.34%
Barren land area	52.48%

**Table 10:** The *R*^2^ of the estimated RFRTM, CNNRTM, and SVMRTM LSTs compared with the MERSI-LL LST and computing time (for each pixel and the study area) for implementing the RFRTM, CNNRTM, and SVMRTM methods during the target period.

Method	Case	*R* ^2^	Computing Time (Dawn and Dusk Conditions Together)
RF	Dusk	0.95	1690 ms (average for each pixel) 11.73 days (for the study area in 10 parallel processing programs)
	Dawn	0.97	
CNN	Dusk	0.95	2540 ms (average for each pixel) 17.62 days (for the study area in 10 parallel processing programs)
	Dawn	0.97	
SVM	Dusk	0.93	1810 ms (average for each pixel) 12.55 days (for the study area in 10 parallel processing programs)
	Dawn	0.94	

**Table 11:** The maximum difference in *R*^2^, MBE, and RMSE between the estimated LST using RF and CNN models (RFRTM LST-CNNRTM LST) over the ground stations during the target period.

Station	Maximum *R*^2^ Difference	Maximum MBE Difference (K)	Maximum RMSE Difference (K)
AR	0.00	0.01	0.02
DM	0.00	−0.02	0.06
DSL	0.01	0.05	−0.07
HZZ	0.01	−0.04	0.07
D66	0.01	0.03	−0.05
D105	0.00	−0.04	0.06
GZ	0.01	0.00	0.00
MQ	0.01	0.03	−0.05
PRD	0.00	−0.02	0.03

## Data Availability

Data are contained within the article.
